# Imaging findings after a total reconstructed breast with autologous fat transfer: what the radiologist needs to know

**DOI:** 10.1093/bjro/tzae010

**Published:** 2024-04-24

**Authors:** Maud E P Rijkx, Esther M Heuts, Janneke B Houwers, Juliette E Hommes, Andrzej A Piatkowski, Thiemo J A van Nijnatten

**Affiliations:** Department of Plastic, Reconstructive, and Hand Surgery, Maastricht University Medical Center+, 6202 AZ, Maastricht, The Netherlands; NUTRIM School for Nutrition, and Translational Research in Metabolism, Maastricht University, 6229 ER, Maastricht, The Netherlands; Department of Surgery, Maastricht University Medical Center+, 6202 AZ, Maastricht, The Netherlands; GROW Research Institute for Oncology and Reproduction, Maastricht University, 6229 ER, Maastricht, The Netherlands; Department of Radiology and Nuclear Medicine, Maastricht University Medical Center+, 6202 AZ, Maastricht, The Netherlands; Department of Plastic, Reconstructive, and Hand Surgery, Maastricht University Medical Center+, 6202 AZ, Maastricht, The Netherlands; NUTRIM School for Nutrition, and Translational Research in Metabolism, Maastricht University, 6229 ER, Maastricht, The Netherlands; Department of Plastic, Reconstructive, and Hand Surgery, Maastricht University Medical Center+, 6202 AZ, Maastricht, The Netherlands; NUTRIM School for Nutrition, and Translational Research in Metabolism, Maastricht University, 6229 ER, Maastricht, The Netherlands; GROW Research Institute for Oncology and Reproduction, Maastricht University, 6229 ER, Maastricht, The Netherlands; Department of Radiology and Nuclear Medicine, Maastricht University Medical Center+, 6202 AZ, Maastricht, The Netherlands

**Keywords:** breast, autologous fat transfer (AFT), breast reconstruction, imaging of the reconstructed breast

## Abstract

Autologous fat transfer (AFT) is an upcoming technique for total breast reconstruction. Consequently, radiological imaging of women with an AFT reconstructed breast will increase in the coming years, yet radiological experience and evidence after AFT is limited.

The surgical procedure of AFT and follow-up with imaging modalities including mammography (MG), ultrasound (US), and MRI in patients with a total breast reconstruction with AFT are summarized to illustrate the radiological normal and suspicious findings for malignancy.

Imaging after a total breast reconstruction with AFT appears to be based mostly on benign imaging findings with an overall low biopsy rate. As higher volumes are injected in this technique, the risk for the onset of fat necrosis increases. Imaging findings most often are related to fat necrosis after AFT. On MG, fat necrosis can mostly be seen as oil cysts. The occurrence of a breast seroma after total breast reconstruction with AFT is an unfavourable outcome and may require special treatment. Fat deposition in the pectoral muscle is a previously unknown, but benign entity. Although fat necrosis is a benign entity, it can mimic breast cancer (recurrence).

In symptomatic women after total breast reconstruction with AFT, MG and US can be considered as first diagnostic modalities. Breast MRI can be used as a problem-solving tool during later stage. Future studies should investigate the most optimal follow-up strategy, including different imaging modalities, in patients treated with AFT for total breast reconstruction.

## Background

**Figure 1.**
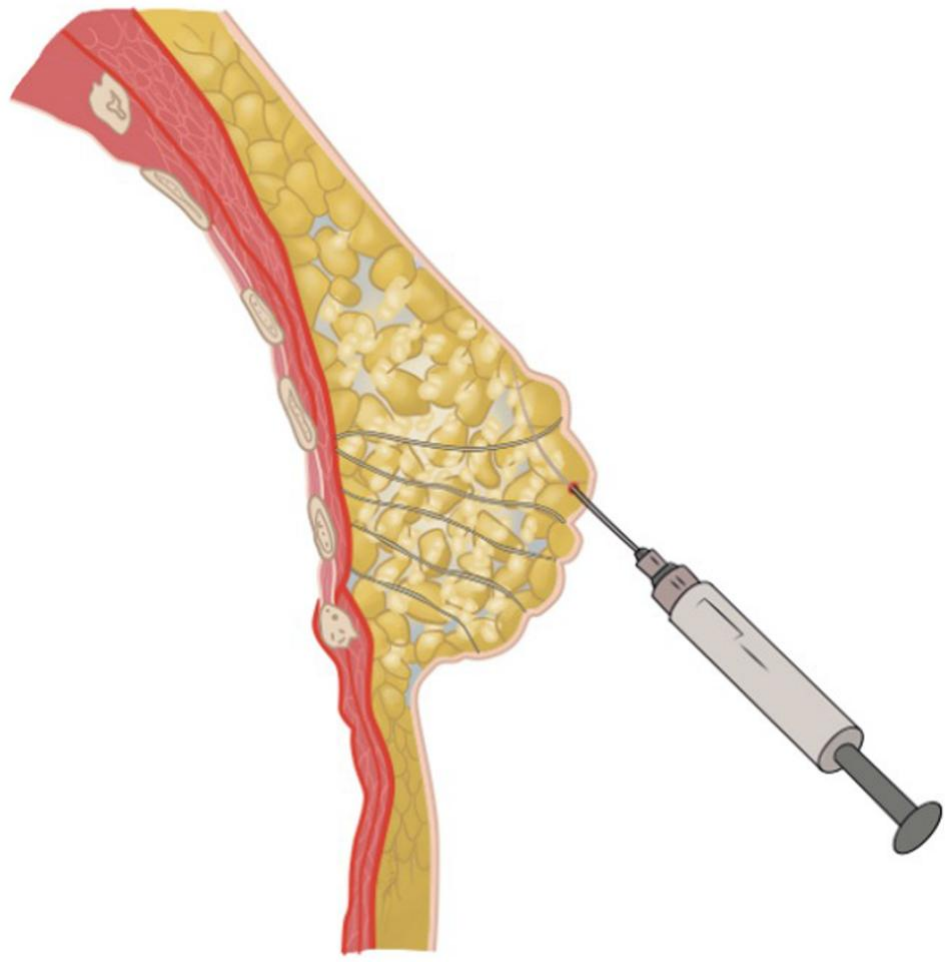
Autologous fat transfer (AFT) as a total breast reconstruction method.

In women who opt for a (prophylactic) mastectomy, breast reconstruction has proven to be a valuable method to restore the breast appearance, the overall satisfaction rate and thereupon the quality of life.^1–3^ Options for postmastectomy breast reconstructions include implant-based reconstructions or autologous breast reconstructions using pedicled or free flaps.^4^ Recently, a new breast reconstruction method, autologous fat transfer (AFT; also known as autologous fat grafting, fat transplantation, fat injection, lipofilling, lipostructuring, lipotransfer, or lipomodelling), has been introduced. This method of reconstruction consists of harvesting autologous fat by liposuction, followed by reinjection of the fat cells into the breast (Figure 1). The use of AFT as a breast reconstruction method was initially limited due to concerns regarding its oncological safety. However, based on recent literature, AFT has been proven to be feasible and oncologically safe.^5–7^

As AFT for total breast reconstruction is increasingly adopted, clinicians need to be aware of the unique challenges involved in AFT patients. One challenge is the evaluation of different imaging modalities after total breast reconstruction with AFT. Based on the fact that AFT as a total breast reconstruction method requires the injection of high volumes of fat (approximately 250 mL per injection site), scar tissue and fat necrosis are likely to occur.^8^ However, since total breast reconstruction with AFT has only been introduced recently, common standard (radiologic) imaging findings following this surgical procedure have not yet been described.

The aim of this comprehensive review is to provide brief information regarding the technical aspects of AFT as a total breast reconstruction method and to give an overview of the common benign findings after AFT, followed by potential suspicious characteristics presented per imaging modality. These include mammography (MG), ultrasound (US), and MRI. The findings are summarized to illustrate the potential benign and suspicious findings for malignancy related to a total breast reconstruction with AFT.

## Surgical technique

**Figure 2.**
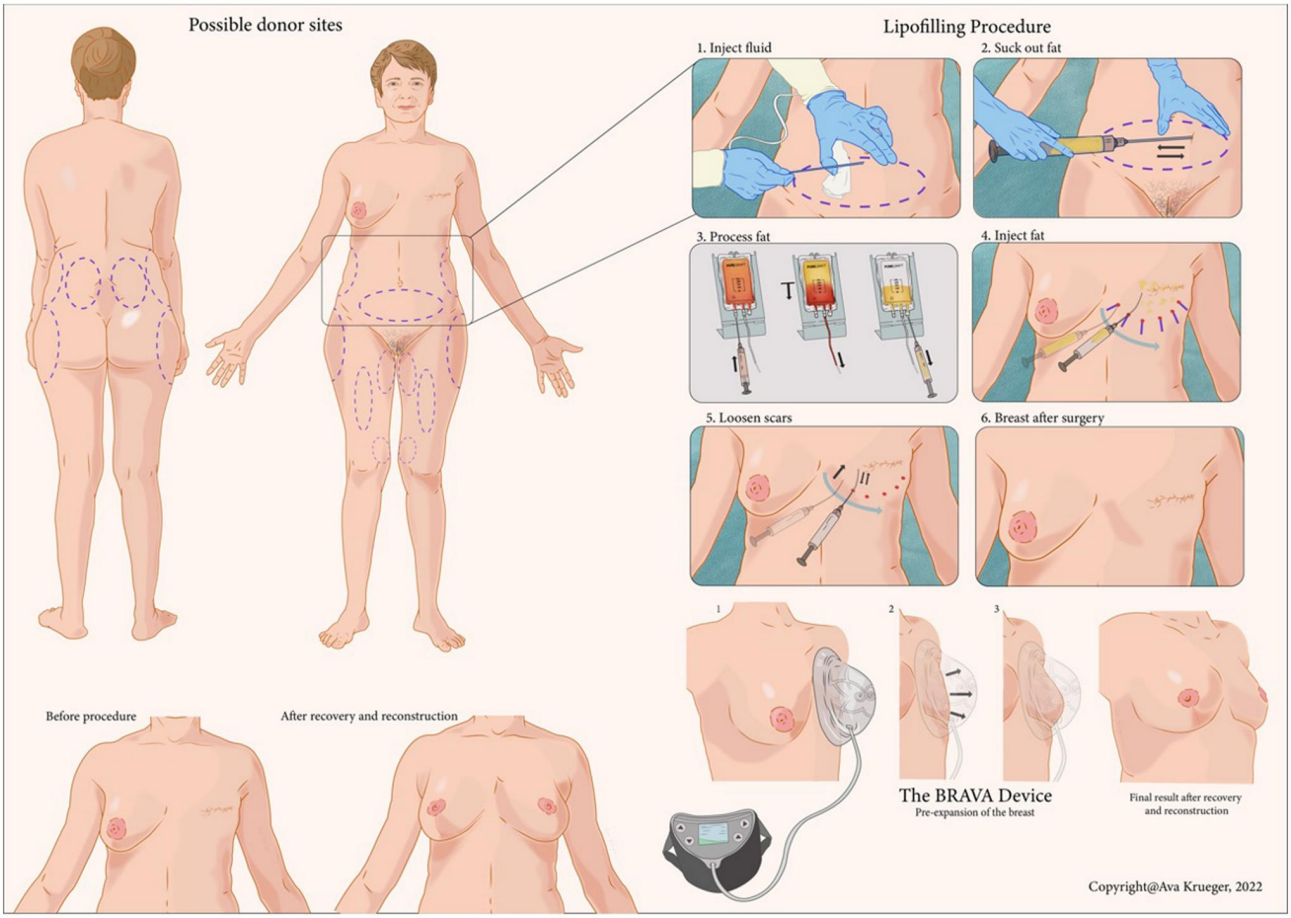
Autologous fat transfer procedure (copyright is held by *JAMA Surgery*, and Ava Krueger, medical and scientific illustrator of this figure).

To reconstruct a total breast with AFT, the following surgical steps are performed as described by Coleman and Khouri (Figure 2).^9–11^ Prior to surgery, a patient is prepared by wearing an external expansion system on the chest including the breast reconstruction area. This external system expands the skin and stimulates the growth of native stromal and vascular elements. At time of surgery, liposuction is performed from predetermined donor sites of the body. Examples of liposuction areas are the belly, thighs, upper legs, or the lower back. Afterwards, the obtained fat is processed. Fat grafting is completed by injecting fat in small aliquots in the separate tissue planes of the breast such as subpectoral, intrapectoral, subdermal, and subcutaneous.

## Benign radiologic findings of a total breast reconstruction with AFT

Imaging after lipofilling to the breast appears to be based mostly on benign imaging findings with an overall low biopsy rate. However, this evidence is limited.^12^ Therefore, the most common benign imaging findings for each benign entity per imaging modality following total breast reconstruction with AFT will be discussed. This includes fat necrosis, fluid collections (seroma), calcifications, and pectoral muscle fat depositions.^13–15^

### Fat necrosis

**Figure 3.**
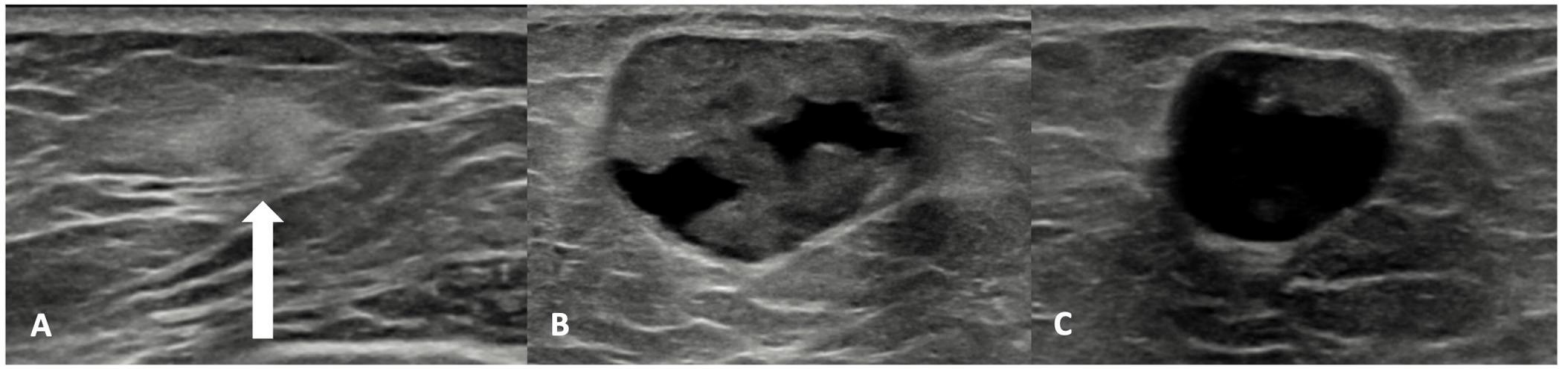
The different stages of fat necrosis. These images are of a 46-year-old female who completed her left total breast reconstruction with AFT 1 year ago. She received a target US. (A) A hyperechogenic area, where a hyperacute phase within the subcutaneous tissue is seen. (B) The inflammatory phase where an oil cyst is starting to form. It also includes a small part of fibrosis in the anterior part of the cyst. This is the third pathophysiological phase. (C) An almost complete oil cyst that only has a small part of fibrosis in the anterior part of the cyst left. Abbreviation: AFT = autologous fat transfer.

Fat necrosis is a sterile, inflammatory process in which adipose tissue cells become necrotic.^16^ It is most common when large amounts of fat are injected, as in total breast reconstruction with AFT.^17^ The pathophysiological appearance of fat necrosis can be divided into 3 consecutive phases: the hyperacute phase, the inflammatory phase, and the fibrotic phase (Figure 3).^16^^,^^18^

**Figure 4.**
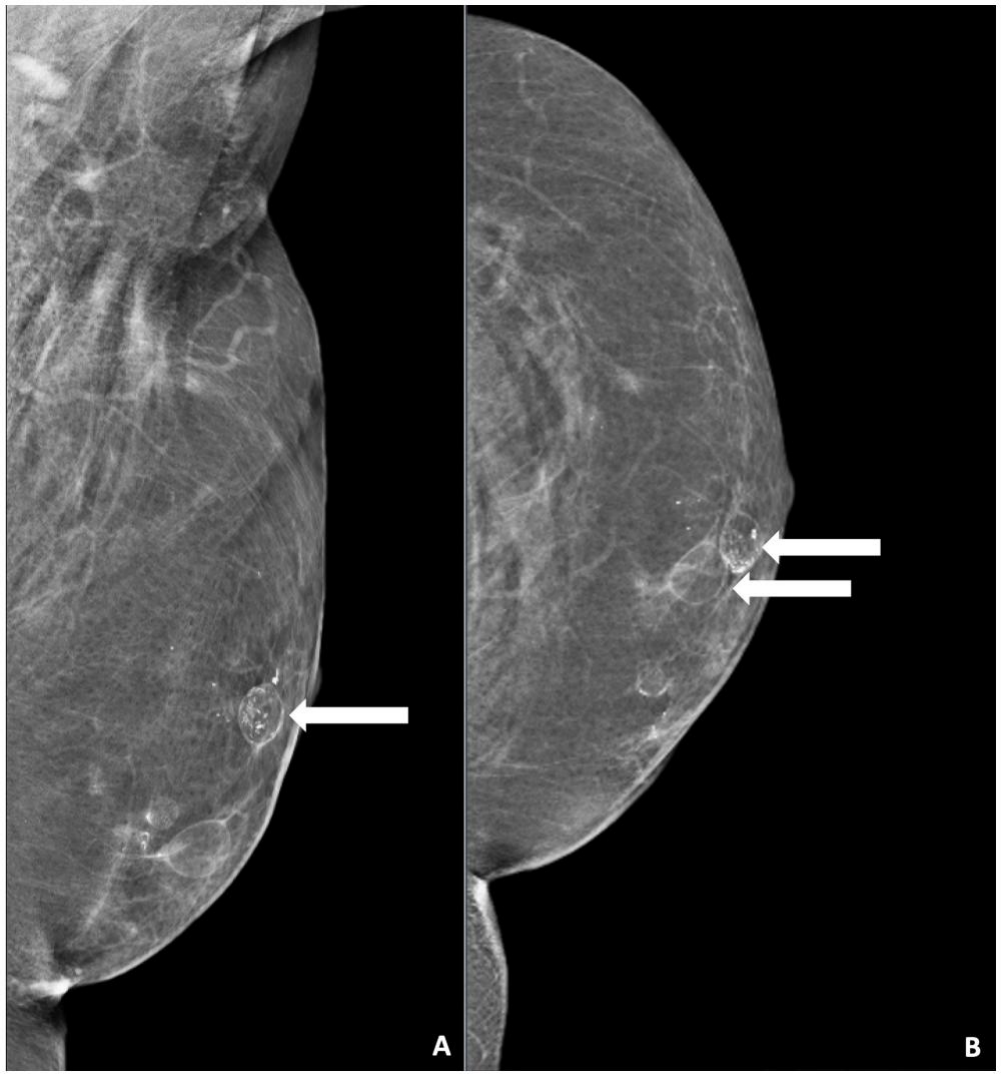
Benign calcifications related to fat necrosis. (A) MLO view of a 46-year-old female patient who completed her left total breast reconstruction with AFT 2 years and 10 months ago. An oval mass with hyperdense membrane can be observed in the former retro areolar region (white arrow), surrounded by multiple calcifications. The mass is corresponding with an oil cyst. (B) Corresponding CC view of the same patient. A total of 2 oil cysts can be appreciated in the former retro areolar region (white arrows). Abbreviations: AFT = autologous fat transfer; MLO = mediolateral oblique.

On MG, fat necrosis can most commonly be seen as oil cysts.^18^ An oil cyst can be recognized as a round radiolucent area or mass and may be associated with either shell like thin or massive, dynamically evolving calcifications, interpreted as BI-RADS 2 (Figure 4).^17^^,^^19^

Oil cysts can evolve in predictable steps and may be accompanied by calcifications. In the early phase, there still is a centre of fat debris present, and the oil cysts may consist of thickened walls with linear and curvilinear surrounded rim calcifications, though rare in uncomplicated cysts. In the end phase, all the fat debris will be replaced by fibrosis which is visible as an irregular spiculated density, a focal dense mass, or an asymmetric density with or without the presence of ring-like dystrophic calcifications in the wall on MG. Typical benign calcifications that are related to fat necrosis are smooth, and round or curvilinear (Figure 4).

Although current experience and evidence on US after AFT is limited, US findings of fat necrosis include the presence of a solid mass or less frequently a cystic mass in combination with an area of increased echogenicity or even architectural distortion of the subcutaneous tissue.^20–22^

Cystic masses with clear margins after a total breast reconstruction with AFT, in combination with the mammographic findings of typical fat necrotic calcifications, can be considered benign. Depending on the stage of fat necrosis, US can differ. In early stage the fat necrosis is hyperechoic or may be heterogeneous with some cystic parts. Even fluid-fluid levels have been observed. Fat necrosis eventually evolves into anechoic cysts, sometimes with rim calcifications presenting on US as a hyperechoic rim, with dorsal acoustic shadowing. According to Parikh et al, if on US an avascular mass is seen at the site of the fat injection, there is a 100% negative predictive value for malignancy and can therefore be considered BI-RADS 2.^23^^,^^24^

**Figure 5.**
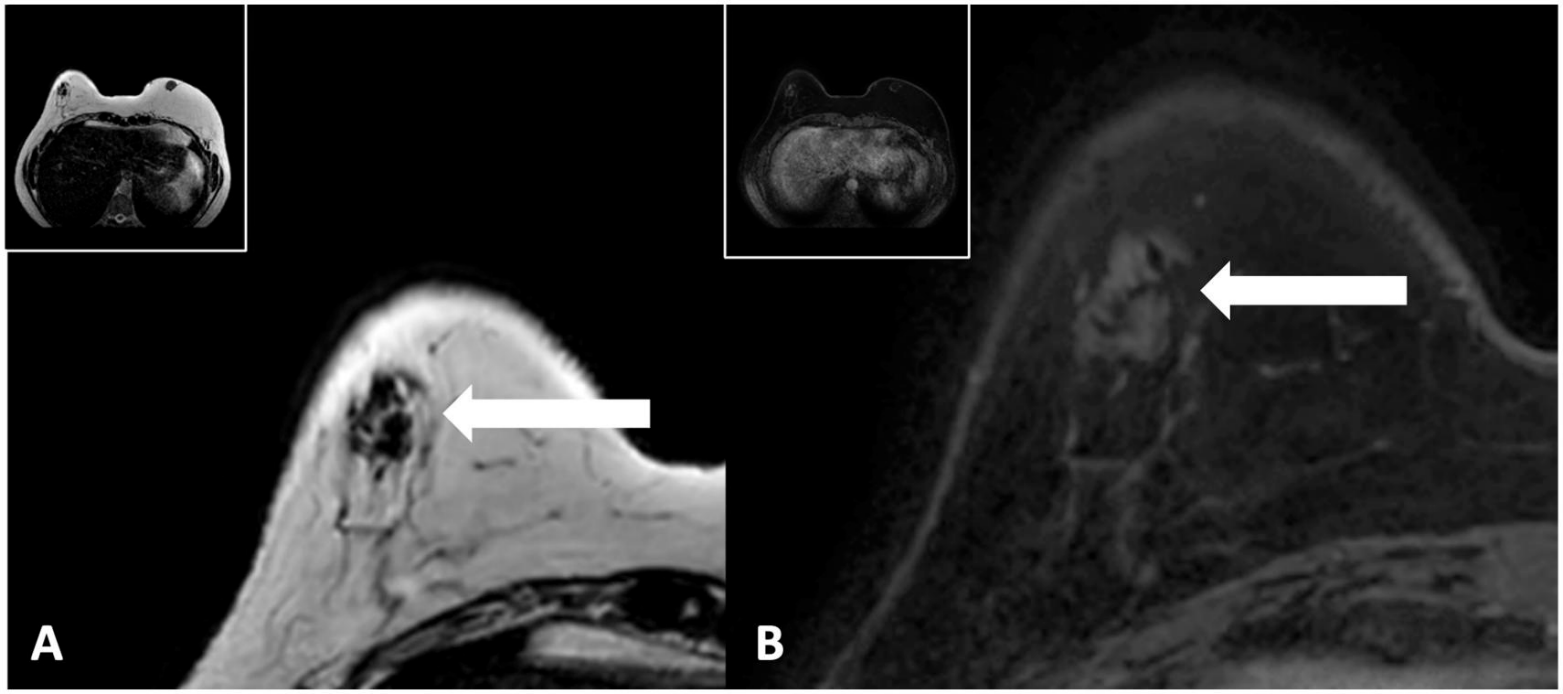
Typical aspects of fat necrosis on MRI. The images are from a 46-year-old female patient that underwent breast MRI 2 years and 10 months after her last AFT. Detailed images of the right breast demonstrate a T2 hypointense lesion (or mass) in the upper-outer quadrant of the right breast (A) without any enhancement on the T1W-sequence after contrast administration (B). Abbreviation: AFT = autologous fat transfer.

On MRI in the AFT reconstructed breast, most signal intensity of the breast is equally to fat, being bright on T1- and T2-sequences, without restriction on diffusion-weighted imaging. Normal lipofilling outcomes may appear as round hypointense findings on fat-suppressed images, without enhancement after gadolinium injection.^17^ However, in the hyperacute and inflammatory phase of fat necrosis, the signal intensity on T1- and T2-sequences can be heterogeneous and being better depicted as hyperintense lesions on T1 fat-suppressed images.^25^^,^^26^ There even may be enhancement after gadolinium injection with variable diffusion restriction and low apparent diffusion coefficient values. Typical aspects of the end stage of fat necrosis are round or oval masses with smooth margins that show hyperintense on T1- (isointense to fat) and T2-weighted images, and hypointense on T1 fat-suppressed images (Figure 5).^20^

### Fluid collections (seroma)

After total breast reconstruction with AFT, fluid collections in the early phase can occur. The differential diagnosis for early-appearing fluid collections in the operated breast includes haematoma, seroma, or abscess. Seroma is an unfavourable outcome after AFT in the breast that may require appropriate treatment and follow-up. It negatively affects a patient by requiring multiple extra clinical visits and may lead to extra mental stress.^27^

**Figure 6.**
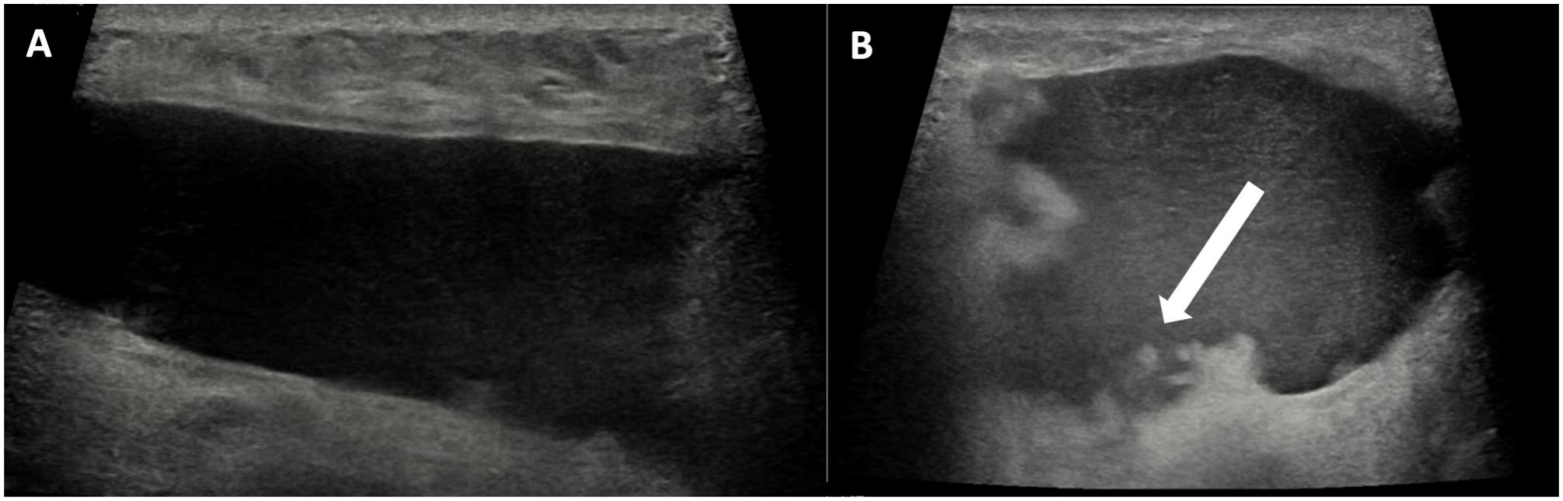
Breast seroma after total reconstruction with AFT on ultrasound. This is an US image of a 53-year-old female patient, 1 month after her second bilateral AFT procedure. Postoperative she suffered from bilateral seroma. (A) An example of an anechogenic fluid collection in the breast. (B) A hypoechogenic fluid collection. The increased echogenity compared to (A) suggests the presence of free-floating fat particles, which may complicate drainage. Abbreviations: AFT = autologous fat transfer; US = ultrasound.

Seromas are generally detected within the first week after surgery. After physical examination by the referring physician, US is performed. In the early phase after surgery, seroma can be identified on US as a heterogenic fluid collection, possibly in combination with a postoperative haematoma. This may require drainage treatment, and after 1 week of follow-up can be considered BI-RADS 2 in the case of consistent findings. Over time thickened nodular margins with internal echoes can occur. Sometimes, if only seroma drainage is not effective, in rare cases, it is possible for a radiologist to consider additional sclerotherapy directly after seroma drainage into the collapsed seroma cavity. Tetracycline can be considered as treatment option.^28^ Important in the treatment of breast seromas after total breast reconstruction with AFT is the size of the aspiration needle. In some cases, it can be possible that within the fluid, loose fat particles are present (Figure 6). These particles can compromise the fine needle aspiration and complicate standard drainage treatment. In these cases, a bigger aspiration needle (5F drain or even bigger) can be necessary. After drainage, weekly evaluation is required to decide if the seroma is in remission or requires repeated drainage treatment. After follow-up, seromas can be considered BI-RADS 2.

**Figure 7.**
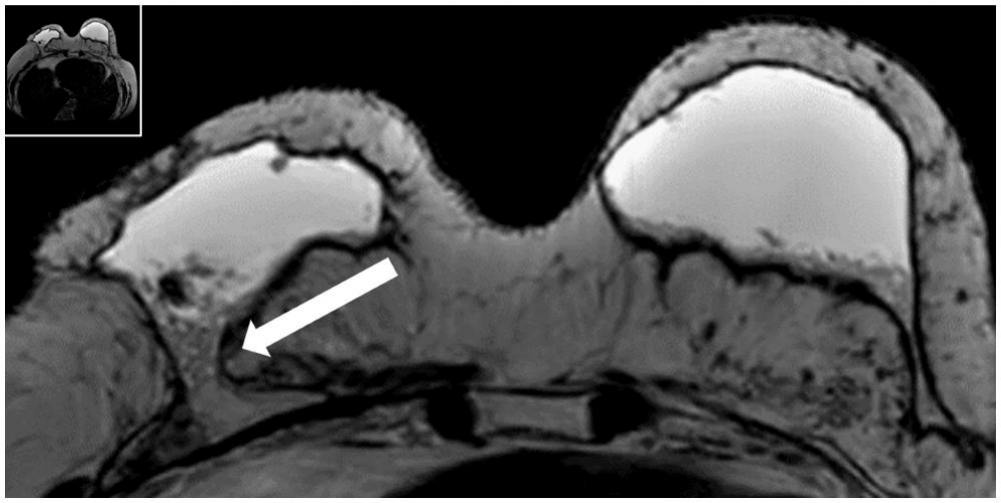
Breast seroma after total breast reconstruction with AFT on MRI. This is a breast MRI of the same 53-year-old patient that had a persistent seroma despite multiple drainage treatments and sclerotherapy. The axial T2-image shows bilateral hyperintense collections. The white arrow demonstrates that the right fluid collection is continuous with the pectoral muscle and a gravity dependent gradient/layering of fluid-debris is seen. Abbreviations: AFT = autologous fat transfer; MRI = magnetic resonance imaging.

If chronic seromas (ie, more than 4 weeks) do not respond to the previously described treatment options with repeated drainage and/or sclerotherapy, additional breast MRI can be performed to identify an underlying cause.^29^ In AFT as a total breast reconstruction method, seromas can potentially involve the pectoral muscle. This can cause ineffective drainage treatment due to remaining fluid in combination with loose fat deposits (Figure 7). On breast MRI, simple seromas appear as uni- or multilocular homogeneous collections, bright on T2- and dark on T1-sequences and considered BI-RADS 2.^25^ Depending on the amount of cellularity and accompanying haemorrhage, the T1-signal intensity may be more hyperintense and the T2 signal intensity becomes more iso- to hypointense. Complicated seromas have heterogeneous signal intensities. If other potential causes are ruled out that can be treated conservatively, complete capsulectomy with quilting sutures of the seroma cavity can be considered by the (plastic) surgeon to achieve complete healing.

### Benign calcifications

Calcifications frequently occur concurrently with fat necrosis.^16^^,^^18^ After AFT, the benign calcification types include coarse, dystrophic, and rim calcifications.^17^^,^^30^ It is important to realize that bilateral, diffuse, and/or symmetric distribution of dystrophic or coarse calcifications, especially in combination with oil cysts and the knowledge of an AFT procedure in the medical history, are benign findings after an AFT procedure and therefore should be considered BI-RADS 2 (Figure 4).

### Fat deposition in the pectoral muscle

**Figure 8.**
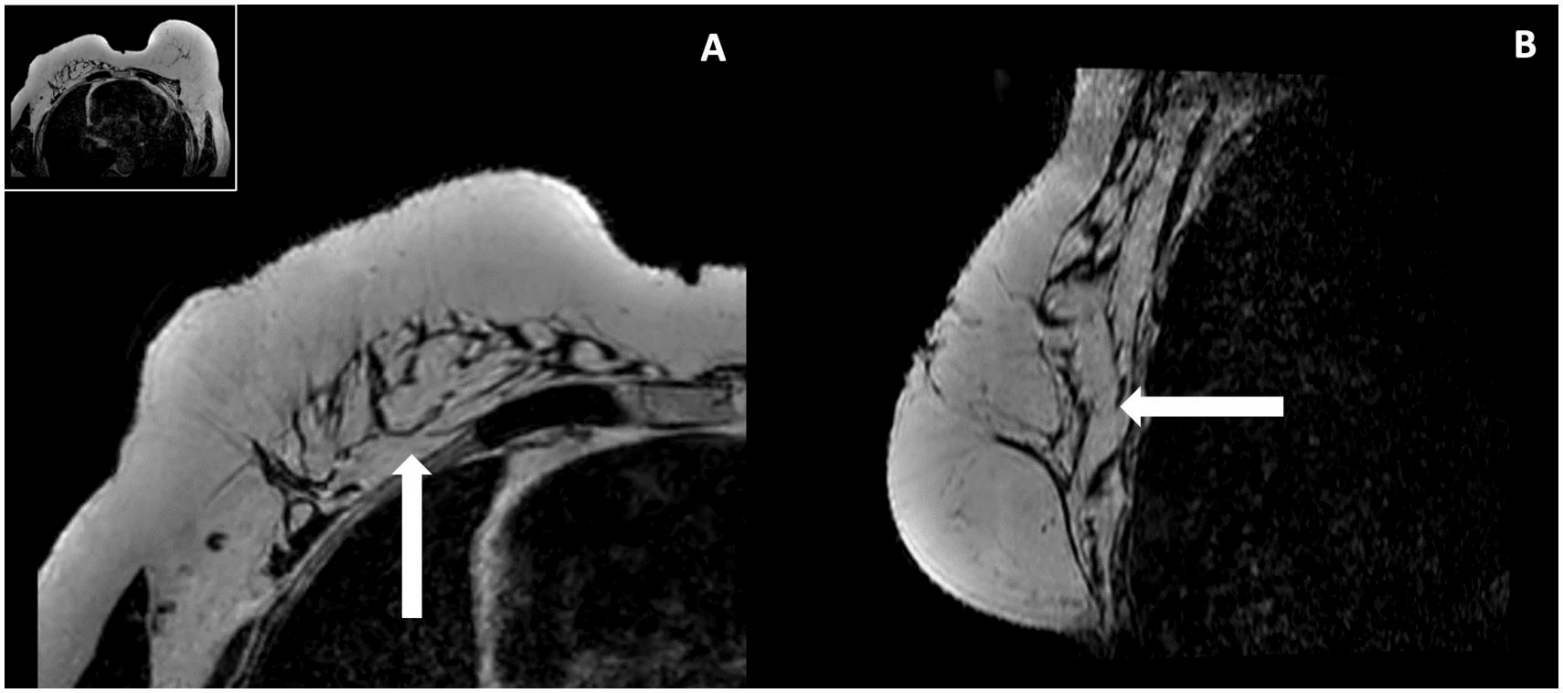
Radiographic change of the pectoral muscle after finishing a total breast reconstruction with AFT. This is a beast MRI of a 68-year-old patient that was 2 years after her last bilateral AFT procedure. (A) The axial T2W image of the right reconstructed breast with fat depositions within the pectoral muscle. (B) The corresponding sagittal view. Abbreviations: AFT = autologous fat transfer; MRI = magnetic resonance imaging; T2W = T2 weighted.

In immediate total breast reconstructions with AFT, fat can be injected under visual control and added into the pectoralis muscle if desired to improve breast volume. However, in AFT as a secondary breast reconstruction, fat is blindly injected by the surgeon within different tissue planes. Although filling up the pectoralis muscle with fat tissue during this procedure may not be the primary intention, it can occur. This happens most often during the first session after mastectomy when there is little (subcutaneous) fat tissue between the skin and the direct underlying pectoralis muscle. Fat in the pectoralis muscle can be seen between the muscle fibres and can be visible on US, MG, and breast MRI. So far, there have been no studies reporting the observation of lipofilling in the pectoralis muscle as part of AFT as a total breast reconstruction method. Figure 8 shows the radiographic change of the pectoral muscle after finishing a total breast reconstruction with AFT on breast MRI.

## Suspicious findings in a total breast reconstruction with AFT

### Mammography

**Figure 9.**
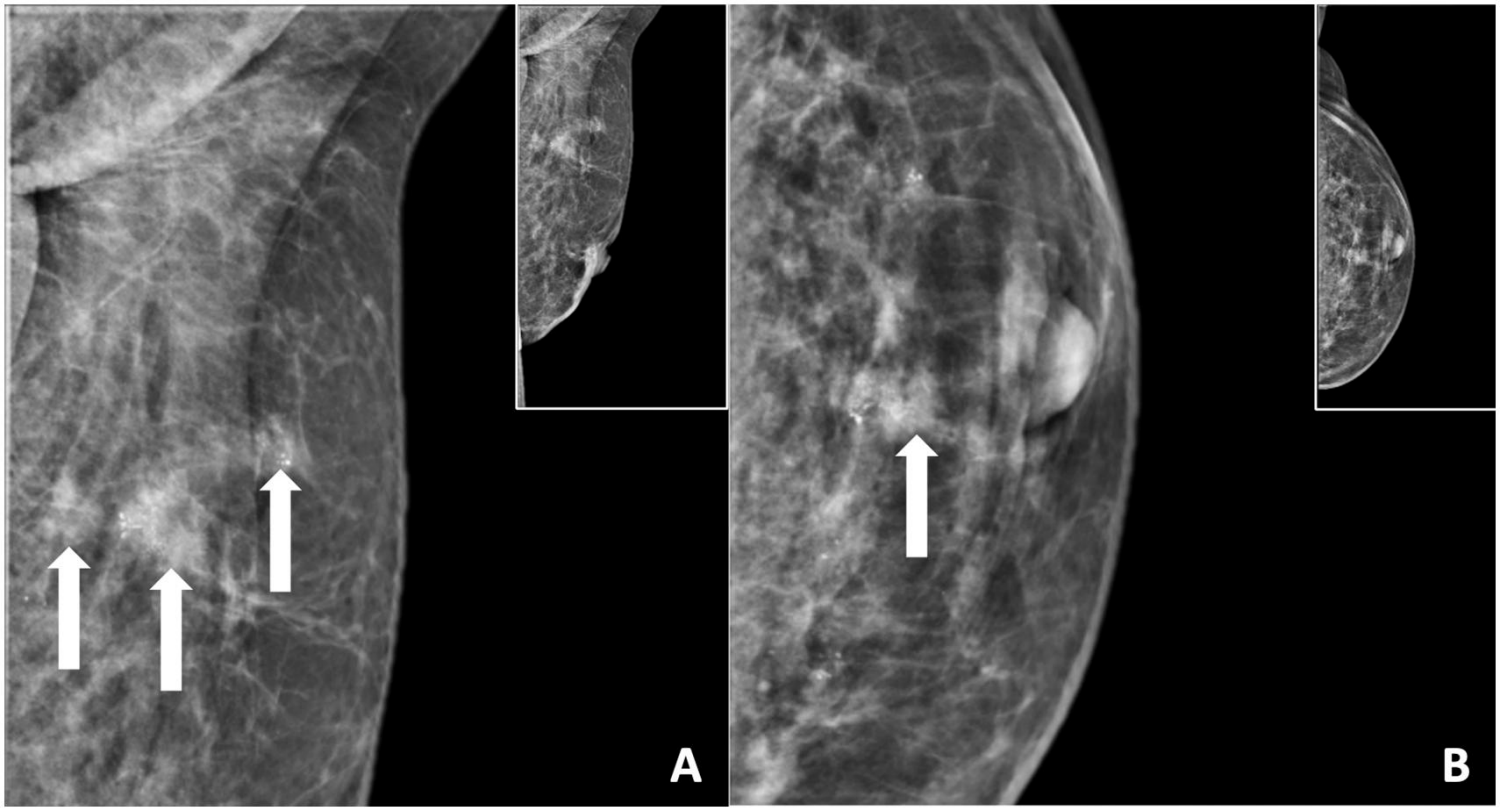
Suspicious findings for malignancy after a total breast reconstruction with AFT on mammography. This is a mammography of a 54-year-old female patient. Her prior medical history includes a DCIS stage 3, treated with a mastectomy and direct breast reconstruction. This mammography was taken 12 months after her final AFT session and shows multiple grouped and clustered pleiomorphic calcifications with a maximum diameter of up to 9 mm in the upper quadrant of the breast (BI-RADS 4). Note in addition the lucent areas within the pectoral muscle, representing pectoral muscle fat depositions. Abbreviation: AFT = autologous fat transfer.

Calcifications on MG cause concern for (recurrent) malignancy if they are branching, rod-like, or angular, after AFT as a total breast reconstruction method.^12^^,^^20^^,^^21^^,^^32^ Clustered calcifications have been observed following AFT in breast augmentations which were unable to be differentiated from breast carcinoma calcifications, necessitating biopsies to rule out malignancy.^31^ The calcifications were only able to be determined benign after biopsy. Although fat necrosis is a benign entity, it can mimic breast cancer through the development of grouped or linear pleomorphic or coarse heterogeneous calcifications (Figure 9). Mammographic suspicious calcifications include grouped amorphous, heterogenous coarse, fine pleomorphic, and fine linear or fine-linear branching and should therefore be considered BI-RADS 4/5 (depending on morphology and distribution pattern), according to the BI-RADS lexicon.^32^

### Ultrasound

It is recognized that US has some limitations regarding differentiation between fat necrosis and lesions suspected of malignancy. One of these limitations is that fat necrosis according to its pathophysiological stage can present differently. In suspicious lesions after a total breast reconstruction with AFT, other US characteristics such as margins, shape, and hypervascularity remain important to evaluate for potential malignancy. Lesions on US with both uncircumscribed margins and vascular internal blood flow signs are predictive for breast cancer (recurrence). Patients should be submitted directly for biopsy to differentiate between breast cancer (recurrence) and fat necrosis.^24^ If there is no mammographic correlation in regard to US findings, or there is an atypical appearing/ill-defined cystic mass, this should be considered at least as a BI-RADS 4 lesion requiring biopsy.

### Breast MRI

**Figure 10.**
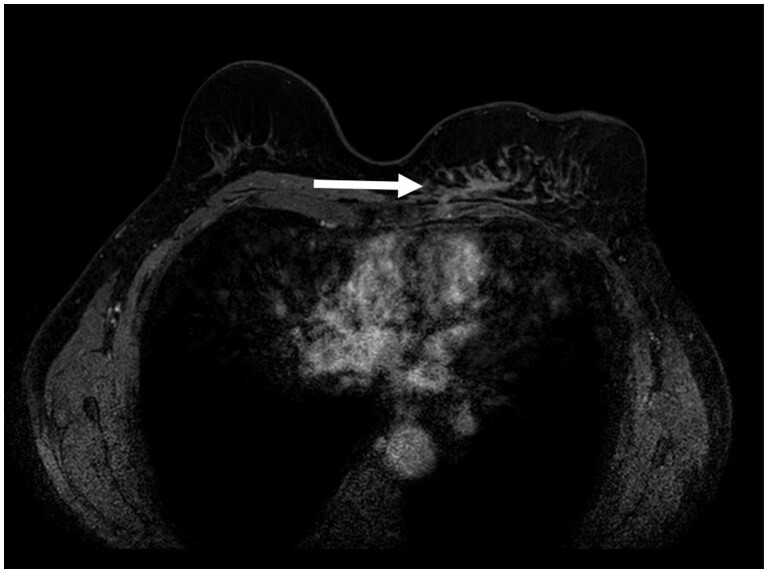
Breast MRI of a 64-year-old woman with a unilateral left-sided total breast reconstruction with AFT, because of a multifocal left-sided invasive carcinoma NST (ER+PR+Her2−) treated with mastectomy. Breast MRI demonstrates an enhancing mass prepectoral on the inner side of the reconstructed breast extending through the pectoral muscle (white arrow). After core-needle biopsy, recurrent breast cancer was confirmed (invasive carcinoma NST, ER+PR+Her2−). Abbreviation: AFT = autologous fat transfer.

Breast MRI has the highest sensitivity for breast cancer detection and is considered an accurate imaging modality for differentiation between benign and suspicious findings after a total breast reconstruction with AFT.^33^ The most important sequence in breast MRI is the contrast-enhanced (T1W) sequence.^34^ The contrast-enhanced T1W-sequence can discriminate whether suspicious findings enhance or not, which is considered one of the key elements for breast MRI.^33^ Mass enhancement or nonmass enhancement, morphologic features, kinetic curve information, and additional findings according to the BI-RADS lexicon will determine whether the radiological finding on breast MRI should be considered suspect for malignancy and require additional biopsy.^32^Figure 10 demonstrates an example case of a patient with recurrent breast cancer after a total breast reconstruction with AFT that underwent MRI.

There is currently no scientific evidence on breast MRI findings in AFT as total breast reconstruction method. The results of breast MRI in patients who have received AFT for total breast reconstruction might serve as a problem-solving tool: if there is no enhancement on the contrast-enhanced T1W-sequence, the need for additional biopsy may be omitted. But, when in doubt after a suspicious breast MRI finding, correlation with MG and/or US can be helpful in the ability to provide additional biopsy to rule out (recurrent) disease. Future studies should collect more scientific evidence regarding the diagnostic performance of breast MRI after AFT for total breast reconstruction.

## Discussion

This is the first comprehensive review describing benign and suspicious radiological findings for malignancy in patients with AFT as a total breast reconstruction method. Current available literature on radiologic implications of fat grafting is primarily based on the use of AFT to restore small contour irregularities after breast-conserving therapy, in addition to other breast reconstruction methods or in breast augmentations. So far, there is no available literature on total breast reconstruction with AFT, which makes this study the first. The critical radiological difference is that in using AFT for the improvement of contour irregularities or breast augmentations, additional fibroglandular tissue is present, while in AFT as a total breast reconstruction method, all normal breast tissue has been removed via mastectomy. Future research on breast imaging after AFT as a total breast reconstruction method is necessary to improve diagnostic assessment in these patients.

In total breast reconstruction with AFT, high (250-350 mL range) reinjection volumes are used to reconstruct an entire breast. In general, 4 procedures are necessary to complete a total breast reconstruction with AFT.^7^ As higher volumes are injected with each procedure, the risk for the onset of fat necrosis increases. Therefore, radiological differentiation between benign and suspicious findings is crucial to avoid unnecessary biopsies. Furthermore, during follow-up, patients can suffer from postoperative seroma. This is an unfavourable outcome in the breast after AFT that can be detected with US and MRI. In postoperative seromas after total breast reconstruction with AFT, loose fat particles can be present within the fluid that require appropriate drainage treatment and follow-up. Lastly, after completion of a total breast reconstruction with AFT, fat depositions within the pectoral muscle can be seen between the muscle fibres and can be visible on US, MG, and breast MRI.

Although most palpable findings after AFT in total breast reconstruction are based on fat necrosis and most radiological findings are benign, breast cancer recurrence after a total breast reconstruction with AFT can occur. At present, according to the American College of Radiology (ACR) appropriateness criteria, there is insufficient evidence for the use of breast MRI as initial imaging method in patients with a palpable lump after (autologous) breast reconstruction.^35^ Breast MRI can be used as a problem solver to differentiate between benign and malignant mammographic and/or sonographic findings. Future studies are needed to determine the necessity of the most optimal follow-up strategy, utilizing a combination of imaging modalities such as MG, US, and breast MRI, in patients treated with AFT as a total breast reconstruction method.

## Conclusion

Autologous fat transfer as total breast reconstruction method is a novel breast reconstructive technique. Consequently, it is important to be aware of the potential benign and suspicious radiological findings for malignancy on MG, US, and MRI. In symptomatic women, MG and US can be considered as first diagnostic modalities. Breast MRI in early phase after AFT can be challenging due to postsurgical enhancement. Future studies should investigate the most optimal follow-up strategy, including different imaging modalities, in patients treated with AFT as a total breast reconstruction method.

## Data Availability

The data that support the findings of this study are available from the corresponding author, Maud E.P. Rijkx, upon reasonable request.
